# Natural brominated phenoxyphenols kill persistent and biofilm-incorporated cells of MRSA and other pathogenic bacteria

**DOI:** 10.1007/s00253-020-10654-4

**Published:** 2020-05-16

**Authors:** Lasse van Geelen, Farnusch Kaschani, Shabnam S. Sazzadeh, Emmanuel T. Adeniyi, Dieter Meier, Peter Proksch, Klaus Pfeffer, Markus Kaiser, Thomas R. Ioerger, Rainer Kalscheuer

**Affiliations:** 1 Institute of Pharmaceutical Biology and Biotechnology, Heinrich Heine University Düsseldorf, Dusseldorf, Germany; 2 Center of Medical Biotechnology, Chemical Biology, University Duisburg-Essen, Duisburg, Germany; 3 Institute of Medical Microbiology and Hospital Hygiene, Heinrich Heine University Düsseldorf, Dusseldorf, Germany; 4 Department of Computer Science, Texas A&M University, College Station, TX USA

**Keywords:** Natural products, Antibiofilm activity, Multidrug resistance

## Abstract

**Abstract:**

Due to a high unresponsiveness to chemotherapy, biofilm formation is an important medical problem that frequently occurs during infection with many bacterial pathogens. In this study, the marine sponge-derived natural compounds 4,6-dibromo-2-(2′,4′-dibromophenoxy)phenol and 3,4,6-tribromo-2-(2′,4′-dibromophenoxy)phenol were found to exhibit broad antibacterial activity against medically relevant gram-positive and gram-negative pathogens. The compounds were not only bactericidal against both replicating and stationary phase–persistent planktonic cells of methicillin-resistant *Staphylococcus aureus* (MRSA) and *Pseudomonas aeruginosa*; they also killed biofilm-incorporated cells of both species while not affecting biofilm structural integrity. Moreover, these compounds were active against carbapenemase-producing *Enterobacter* sp. This simultaneous activity of compounds against different growth forms of both gram-positive and gram-negative bacteria is rare. Genome sequencing of spontaneous resistant mutants and proteome analysis suggest that resistance is mediated by downregulation of the bacterial EIIBC phosphotransferase components *scrA* and *mtlA* in MRSA likely leading to a lower uptake of the molecules. Due to their only moderate cytotoxicity against human cell lines, phenoxyphenols provide an interesting new scaffold for development of antimicrobial agents with activity against planktonic cells, persisters and biofilm-incoporated cells of ESKAPE pathogens.

**Key points:**

*• Brominated phenoxyphenols kill actively replicating and biofilm-incorporated bacteria.*

*• Phosphotransferase systems mediate uptake of brominated phenoxyphenols.*

*• Downregulation of phosphotransferase systems mediate resistance.*

**Electronic supplementary material:**

The online version of this article (10.1007/s00253-020-10654-4) contains supplementary material, which is available to authorized users.

## Introduction

*Staphylococcus aureus* is a gram-positive bacterium, which is part of the human skin flora but most commonly known as an opportunistic pathogen causing different life-threatening diseases such as endocarditis or toxic shock syndrome (Boswihi and Udo 2018; Haysom et al. 2018; Lowy 1998; Wolk et al. 2009). The most prominent member in the group of pathogenic staphylococci is methicillin-resistant *S. aureus*, commonly known as MRSA. It first occurred almost 80 years ago in the 1940s just a few years after the introduction of penicillin, but about 20 years before methicillin was first used as an antibiotic (Harkins et al. 2017; Jevons 1961; Kirby 1944; Rammelkamp and Maxon 1942). A single mutation in the gene *mecA*, located on the staphylococcal cassette chromosome *mec* (SCC*mec*) coding for a low-binding affinity penicillin-binding protein (PBP), is the source of MRSA’s resistance not only to methicillin but to plenty of members of ß-lactam antibiotics (Hartman and Tomasz 1984; Lakhundi and Zhang 2018; Matsuhashi et al. 1986). This example shows that antimicrobial resistance (AMR) and cross-resistance can emerge very fast and can spread among a population via mobile genetic elements like SCC*mec*. Nowadays, clinical isolates of MRSA frequently show multidrug resistance against several classes of antibiotics like macrolides, lincosamides, fluoroquinolones and aminoglycosides (Chambers and Deleo 2009; Foster 2017; Jayaweera and Kumbukgolla 2017; Kaur and Chate 2015). In addition to MRSA, multidrug resistance (MDR) is also widespread among other members of the so-called ESKAPE pathogens such as the gram-negative bacterium *Enterobacter cloacae*. Recently, we have isolated and characterised clinically relevant MDR *E. cloacae* strains harbouring the metallo-β-lactamase (MBL) bla_GIM-1_ (German imipenemase-1) (Wendel et al. 2013; Wendel et al. 2016), conferring resistance against imipenem and meropenem, and which are additionally resistant against aztreonam, ciprofloxacin, trimethoprim-sulfamethoxazole, tigecycline and chloramphenicol.

MRSA, like other bacteria, is able to change its growth behaviour. Persistence and biofilm formation render bacteria phenotypically resistant against most antibiotics, despite being genetically identical to their replicating counterparts. Persistence is widely spread in bacteria and known for both gram-positive and gram-negative bacteria alike (Barraud et al. 2013; Bigger 1944; Stewart and Rozen 2012). The state of persistence is mainly characterised by a dramatically slowed metabolism as well as the stop of cell division, replication of their genome or protein synthesis. Via different mechanisms, such as toxin-antitoxin systems, bacteria are able to adapt very quickly to environmental changes like antibiotic treatment and consequently switch to persistence (Balaban 2011; Balaban et al. 2004; Conlon et al. 2016). Biofilms are often the reason for the resurgence of infections. Surfaces like plastic catheters or prosthetic joints provide an appealing scaffold for biofilm-forming bacteria like *S. aureus* and *Pseudomonas aeruginosa*, which are two of the most common biofilm-forming bacteria. Biofilms are composed of replicating and persistent cells, which are surrounded by extracellular polymeric substances (EPSs) such as eDNA, proteins and polysaccharides, forming a physical barrier to protect bacteria from antibiotics or other harmful abiotic and biotic stressors (Hoiby et al. 2010; Flemming 2016). Therefore, almost every antibiotic in clinical use, such as broad spectrum penicillins as the most prescribed antibiotic class in 2015, is inactive against persisters and especially biofilm-incorporated cells (Klein et al. 2018). Both growth forms are able to survive antibiotic treatment with up to 1000-fold of the minimal inhibitory concentrations (MICs) against actively growing cells, and surgical removal of infected tissues and devices is often the last choice (Barraud et al. 2013; Mermel et al. 2009).

**Fig. 1:**
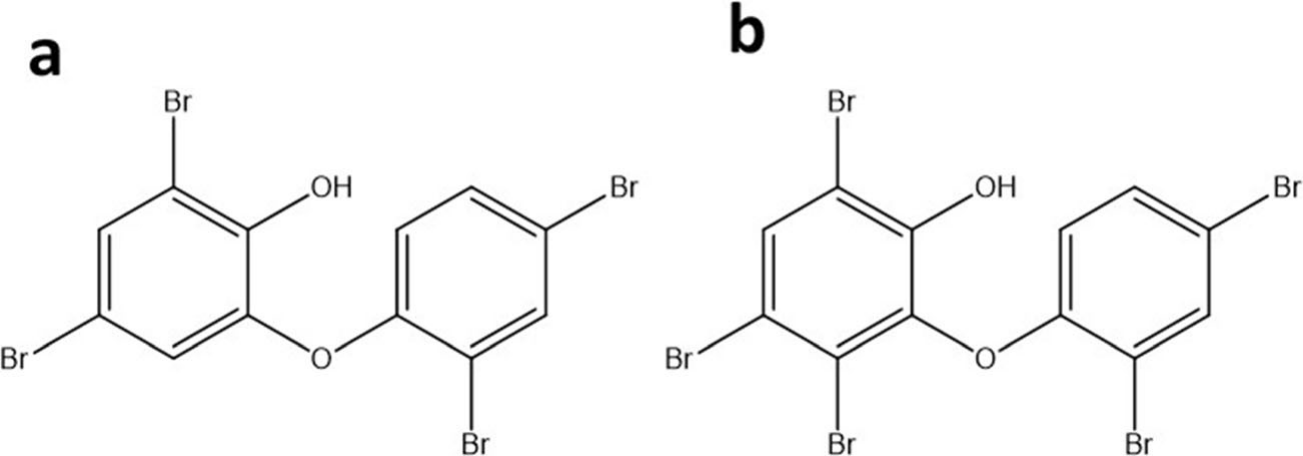
Structures of **a** 2-bromo-PP and **b** 3-bromo-PP. Both molecules were isolated from the marine sponge *Dysidea granulosa* and are highly active against ESKAPE pathogens like MRSA. The difference in both molecules is a single bromide atom at position 3 in the phenol moiety

To address the growing problem of AMR, it is essential to find and develop new lead structures and antibiotics aiming at new targets in the bacterial cell. Several approaches have been used to tackle the problems of rising AMR, persistence and biofilm growth, unfortunately with little success to challenge bacteria in all growth forms simultaneously (Allison et al. 2011; Prax et al. 2016; Zipperer et al. 2016). A rich source for new lead structures is natural products (reviewed in (van Geelen et al. 2018)). Here, we investigated two structurally related compounds isolated from the marine sponge *Dysidea granulosa*, which was collected in the Andaman Sea (Thailand) in 2007. The compounds, 4,6-dibromo-2-(2′,4′-dibromophenoxy)phenol (referred to as 2-bromo-PP) and 3,4,6-tribromo-2-(2′,4′-dibromophenoxy)phenol (referred to as 3-bromo-PP) are both brominated phenoxyphenols (PPs) and only differ in the additional bromide atom in 3-bromo-PP at position 3 in the phenol moiety (Fig. 1). Both compounds are active against replicating, persistent and biofilm-incorporated cells of gram-positive and gram-negative bacteria and show a promising therapeutically window for further investigation.

## Material and methods

### Bacterial strains, plasmids and growth conditions

Strains and plasmids used in this study are listed in Tables S1 and S2, respectively. Cultures were, if not stated differently, incubated at 37 °C with aeration and shaking (150 rpm) in Mueller-Hinton broth (MHB). Bacteria were freshly incubated at the next morning from an overnight pre-culture for actively growing bacteria to an OD_600 nm_ of approx. 0.6. To ensure persistent growth form, the bacteria were grown overnight until a density at OD_600 nm_ > 5.0 was obtained. For biofilm growth, some colonies from a Mueller-Hinton agar (MHA) plate were picked and resuspended in MHB. Cell density has been adjusted to OD_600 nm_ = 0.8, and cells were sown into a flat-bottom 96-well plate to be incubated statically at 37 °C with aeration. Studies were mainly performed with *S. aureus* Mu50.

### Compounds

2-Bromo-PP and 3-bromo-PP were initially isolated from an undescribed *Dysidea* sp. from Satawan Atoll, Chuuk State, Federal States of Micronesia (Fu and Schmitz 1996). The compounds have been reisolated from the marine sponge *Dysidea granulosa* harvested in the Andaman Sea in 2007 and were identified based on their NMR and MS spectroscopic data and comparison with the literature. The molecules are now part of a compound library of the Institute of Pharmaceutical Biology and Biotechnology of the Heinrich Heine University Düsseldorf, Germany. The compounds were freshly prepared as 10 mM stock in DMSO and stored at − 20 °C until further use.

### MIC determination

The minimal inhibitory concentration (MIC) has been determined according to the CSLI guidelines (CLSI 2012). Briefly, in a sterile polystyrene U-bottom 96-well plate, a 2-fold serial dilution ranging from 100 to 0.78 μM of testing compounds has been prepared in 50 μL MHB. A freshly inoculated *S. aureus* culture was grown until OD_600 nm_ of approx. 0.6 and diluted to 10^6^ CFU/mL in MHB. Finally, 50 μL of the latter cell suspension was added to each well and then incubated statically for 18–24 h at 37 °C. MIC was determined using BacTiterGlo ATP assay (Promega, Madison, WI, USA) and Tecan Infinite F200 Pro (Tecan, Männedorf, Switzerland). Moxifloxacin and DMSO were used as positive and negative control, respectively. Sublethal concentrations of colistin (0.1 μM) were used in some experiments to increase the permeability of the gram-negative cell envelope for compound testing. For *E. cloacae*, a broth microdilution assay was performed using a serial 2-fold dilution in 96-well flat-bottomed plates (Falcon, BD Bioscience, Heidelberg) of both PPs in broth LB medium (Roth, Karlsruhe) at a concentration range of 50–0.024 μM.

### Antipersister activity assay

Sterile U-bottom 96-well polystyrene plates were used for the assay. The wells were prepared with a final volume of 50 μL phosphate-buffered saline (PBS, NaCl = 137 mM, KCl = 2.7 mM, NaHPO_4_ = 10 mM, KH_2_PO_4_ = 1.8 mM, pH = 7.4) containing a 2-fold serial dilution of compounds ranging from 100 to 0.78 μM. *S. aureus* was cultivated as described above and washed three times with PBS before adjusting the cell density to approx. 2 × 10^7^ cells/mL (OD_600 nm_ = 0.08). To the prepared 96-well plates, 50 μL of the cell suspension was added to each well. The plate was statically incubated for 24 h at 37 °C.

Viability of bacterial cells was estimated employing the Resazurin dye reduction assay. Briefly, 10 μL of a 100 μg/mL Resazurin solution was added to each well and were resuspend carefully. After several hours of incubation at 37 °C, the cells were inactivated with 10% formalin solution. Finally, the fluorescence was quantified in a plate reader with 535-nm excision and 590-nm emission wavelengths. Moxifloxacin, DMSO and NH125 were used as negative and positive controls, respectively.

### Antibiofilm activity assay

Sterile flat-bottom 96-well polystyrene plates were used for the assay. Some *S. aureus* colonies were picked from an MHA plate and resuspend in MHB + 10% glucose, and cell density was adjusted to 10^8^ cells per mL. One hundred microlitres of cell suspension was added to each well, and the plate was incubated statically for 24 h to induce growth and maturation of biofilms. After 24 h, the wells were washed three times with PBS to remove planktonic cells, and compounds were added to the wells to check for antibiofilm activity. To check for inhibition of biofilm formation, compounds were added immediately after adding of cells to the wells in sublethal concentrations. Lysostaphin, moxifloxacin and DMSO served as positive and negative controls, respectively.

Viability was measured as described above utilizing the Resazurin reduction assay. Biofilm formation was measured using crystal violet staining as described elsewhere (Christensen et al. 1985; Stepanovic et al. 2007). Briefly, biofilms were washed three times with PBS and allowed to air dry before 0.1% (*v*/*v*) crystal violet solution (Waldeck GmbH & Co., Münster, Germany) was added to each well. The biofilms were stained for 15 min at room temperature (RT). Afterwards, the plates were washed three times again with PBS, and 30% acetic acid was added for 30 min at RT to solubilise the dye. Finally, 100 μL was transferred to a fresh 96-well plate to measure absorption at 600 nm with a plate reader.

Additionally, biofilm viability was quantified by CFU plating. Biofilms were pregrown as described above and treated for 24 h with 4× minimal biofilm inhibitory concentrations as calculated from the Resazurin reduction assay. Biofilms were dispersed by pipetting followed by sonification in a water bath. Dilutions were plated on Mueller-Hinton agar plates and incubated overnight at 37 °C.

### Killing kinetics

*S. aureus* cells were freshly incubated in MHB until an OD_600 nm_ of approx. 0.6 was reached. Next, the cells were diluted to 10^6^ CFU/mL in 5 mL MHB. Then compounds were added to the cultures as follows: 2-bromo-PP 0.78 μM (4× MIC), 3-bromo-PP 0.39 μM (4× MIC), moxifloxacin 20 μM (2× MIC) and DMSO 50 μM. At certain time points (0, 0.5, 1, 3, 6, 8, 24 h), 100 μL was removed from the culture, diluted and plated onto MHA plates. After 24-h incubation, colonies were counted and viability (expressed as colony-forming units per mL (CFU/mL)) was calculated considering the dilution factor.

For persister killing kinetics, cells were grown overnight until a density of OD_600 nm_ > 5.0 was reached and then washed three times with PBS. Next, cell density was adjusted to 10^8^ CFU/mL in 5 mL PBS. Compounds were added as follows: 2-bromo-PP 12.5 μM (4× MIC), 3-bromo-PP 6.25 μM (4× MIC), moxifloxacin 100 μM, DMSO 50 μM and NH125 10 μM. Analysis was performed as described above.

### Quantitative Real-Time PCR

Quantitative real-time PCR (qPCR) was performed as described (Lewis and Rice 2016). Briefly, cells were lysed using bead beating in a tissuelyser Precellys 24 (Bertin Instruments, Montigny-le-Bretonneux, France). RNA was isolated using the Qiagen RNeasy kit (Qiagen, Hilden, Germany), and quality was checked with an RNA 6000 Nano Chip in a Bioanalyser (Agilent, Santa Clara, CA, USA) performed by the BMFZ in Duesseldorf. cDNA was synthesised using SuperScript IV First-Strand Synthesis kit (ThermoFisher, Meerbusch, Germany). qPCR was performed using GoTaq qPCR Master Mix from Promega. The experiment was performed as given in the manufacturer’s protocol. As template, 5 μL of 1:10 diluted cDNA samples was used. Results have been normalised to 16S rRNA using the Livak method. All experiments have been performed in triplicates.

### Cytotoxicity assay

Human cell lines were grown in their respective growth medium. Cells have been counted using a haemocytometer, and cell density was adjusted to 10^6^ cells/mL. A 2-fold serial dilution was prepared in 96-well U-bottom plates, and cells were sown into the wells to a final density of 5 × 10^5^ cells/well. After 2 days at 37 °C and 5% CO_2_, 10 μL of a 100 μg/mL Resazurin dye solution was added to each well and was incubated for several hours. The reaction was inactivated with 100 μL of a 10% formalin solution per well. Finally, the fluorescence was quantified in a plate reader with 535-nm excision and 590-nm emission wavelengths. Cell lines are shown in Table S3.

### Fluorescence microscopy

Biofilms were pre-grown as described above. Alcian blue staining was performed to stain the EPS. A 1% Alcian blue staining solution was prepared in 3% glacial acetic acid. Staining solution was added to the biofilm in sufficient amount to cover the whole biofilm and incubated for 30 min at RT. Next, the biofilm was washed twice with PBS, and 200 μL 10% formalin solution was added. Fluorescence microscopy was performed using a Nikon Eclipse TS100 fluorescence microscope.

### Sample clean-up for LC-MS

After in-solution digestion (ISD) peptides were desalted on home-made C18 StageTips (Rappsilber et al. 2007). Briefly, the peptide solution was passed over the MeOH pre-conditioned and 0.5% formic acid (FA) equilibrated StageTip. Immobilised peptides were then washed twice with 0.5% (*v*/*v*) FA. Washed peptides were eluted from the StageTips with 80% (*v*/*v*) ACN and 0.5% (*v*/*v*) FA and dried using a vacuum concentrator (Eppendorf). Before LC-MS, peptide samples were resuspended in 10 μL 0.1% (*v*/*v*) FA.

### LC-MS/MS

LC-MS/MS experiments were performed on an Orbitrap Elite instrument (Michalski et al. 2012) (Thermo) that was coupled to an EASY-nLC 1000 liquid chromatography (LC) system (Thermo). The LC was operated in the one-column mode. The analytical column was a fused silica capillary (75 μm × 45 cm) with an integrated PicoFrit emitter (15 μm, New Objective) packed in-house with Reprosil-Pur 120 C18-AQ 1.9-μm resin (Dr. Maisch). The analytical column was encased by a column oven (Sonation) and attached to a nanospray flex ion source (Thermo). The column oven temperature was adjusted to 45 °C during data acquisition. The LC was equipped with two mobile phases: solvent A (0.1% FA, in water) and solvent B (0.1% FA in acetonitrile (ACN)). All solvents were of UPLC grade (Sigma). Peptides were directly loaded onto the analytical column with a maximum flow rate that would not exceed the set pressure limit of 980 bar (usually around 0.5–0.6 μL/min). Peptides were subsequently separated on the analytical column by running a 140 min gradient of solvent A and solvent B at a flow rate of 300 nL/min (gradient: start with 7% B; gradient 7 to 35% B for 120 min; gradient 35–100% B for 10 min and 100% B for 10 min). The mass spectrometer was operated using Xcalibur software, Thermo Fischer Scientific, UK (version 2.2 SP1.48) and was set in the positive ion mode. Precursor ion scanning was performed in the Orbitrap analyser (FTMS (Fourier transform mass spectrometry)) in the scan range of *m*/*z* 300–1800 and at a resolution of 60,000 with the internal lock mass option turned on (lock mass was 445.120025 *m*/*z*, polysiloxane) (Olsen et al. 2005). Product ion spectra were recorded in a data-dependent fashion in the ion trap (ITMS) in a variable scan range and at a rapid scan rate. The ionization potential (spray voltage) was set to 1.8 kV. Peptides were analysed using a repeating cycle consisting of a full precursor ion scan (1.0 × 10^6^ ions or 50 ms) followed by 15 product ion scans (1.0 × 10^4^ ions or 100 ms), where peptides are isolated based on their intensity in the full survey scan (threshold of 500 counts) for tandem mass spectrum (MS2) generation that permits peptide sequencing and identification. Collision-induced dissociation (CID) energy was set to 35% for the generation of MS2 spectra. During MS2 data acquisition, dynamic ion exclusion was set to 120 s with a maximum list of excluded ions consisting of 500 members and a repeat count of one. Ion injection time prediction, preview mode for the FTMS (the orbitrap), monoisotopic precursor selection and charge state screening were enabled. Only charge states higher than 1 were considered for fragmentation.

### Peptide and protein identification using MaxQuant and Perseus

RAW spectra were submitted to an Andromeda (Cox et al. 2011) search in MaxQuant (version 1.5.3.30) using the default settings (Cox and Mann 2008). Label-free quantification was activated (Cox et al. 2014). MS/MS spectra data were searched against the Uniprot *Staphylococcus aureus* strain Mu50 reference proteome database (UP000002481_158878.fasta; 2714 entries; downloaded 23.02.2017). All searches included a contaminant database (as implemented in MaxQuant, 245 sequences). The contaminant database contains known MS contaminants and was included to estimate the level of contamination. Andromeda searches allowed oxidation of methionine residues (16 Da), acetylation of the protein N-terminus (42 Da) as dynamic modifications and the static modification of cysteine (57 Da, alkylation with Iodoacetamide). Enzyme specificity was set to “Trypsin/P”. The instrument type in Andromeda searches was set to Orbitrap, and the precursor mass tolerance was set to ± 20 ppm (first search) and ± 4.5 ppm (main search). The MS/MS match tolerance was set to ± 0.5 Da. The peptide spectrum match FDR and the protein FDR were set to 0.01 (based on target-decoy approach). Minimum peptide length was 7 amino acids. For protein quantification, unique and razor peptides were allowed. Modified peptides with dynamic modifications were allowed for quantification. The minimum score for modified peptides was 40. Further data analysis and filtering of the MaxQuant output were done in Perseus v1.5.5.3 (Tyanova et al. 2016). MS/MS counts were loaded into the matrix from the proteinGroups.txt file and potential contaminants as well as reverse hits; hits only identified by site and protein groups with less than 2 identified unique peptides were removed. For the statistical calculation samples, technical replicates were grouped in categorical groups and filtered. Only those protein groups were kept that contained three valid values in a minimum of one categorical group. The missing values in the remaining protein groups were then imputed and the *t* test perfomed (number of randomizations 250; initial FDR 0.05 and S0 0.1).

### Data availability

The mass spectrometry proteomic data have been deposited to the ProteomeXchange Consortium via the PRIDE (Vizcaino et al. 2016) partner repository (https://www.ebi.ac.uk/pride/archive/) with the dataset identifier PXD016373.

### Genome sequencing

DNA was extracted from individual colonies using a protocol from Krausz and Bose (Krausz and Bose 2016). The samples were prepared for sequencing using the standard Illumina whole-genome sample preparation kit (Illumina, Inc.; San Diego, CA) and sequenced on an Illumina HiSeq instrument. Paired-end reads with a read length of 150 bp were collected. The mean depth of coverage ranged from 165 to 390. Genome sequences for the isolates were assembled using a comparative assembly approach (Ioerger et al. 2010). Reads were mapped to the genome sequence of the parent *S. aureus* strain (either ATCC 25923 or ATCC 700699, GenBank accession numbers CP009361.1 and NC_002758.2) using BWA v0.7.12 (Li and Durbin 2009), and insertions and deletions (indels) were identified using local contig building. Polymorphisms were identified by aligning each genome to the reference sequence (using MUMmer v3.20 (Kurtz et al. 2004)) and tabulating single-nucleotide polymorphisms (SNPs) and indels according to the following criteria: coverage by at least 10 reads, and not heterogeneous (≥ 70% conversion to the nonreference nucleotide). SNPs in repetitive regions were also filtered out (defined as sites for which an overlapping 35-bp window matched a sequence elsewhere in the genome with at most 2 mismatches).

## Results

### In vitro antibacterial activity of 2-bromo-PP and 3-bromo-PP

**Fig. 2:**
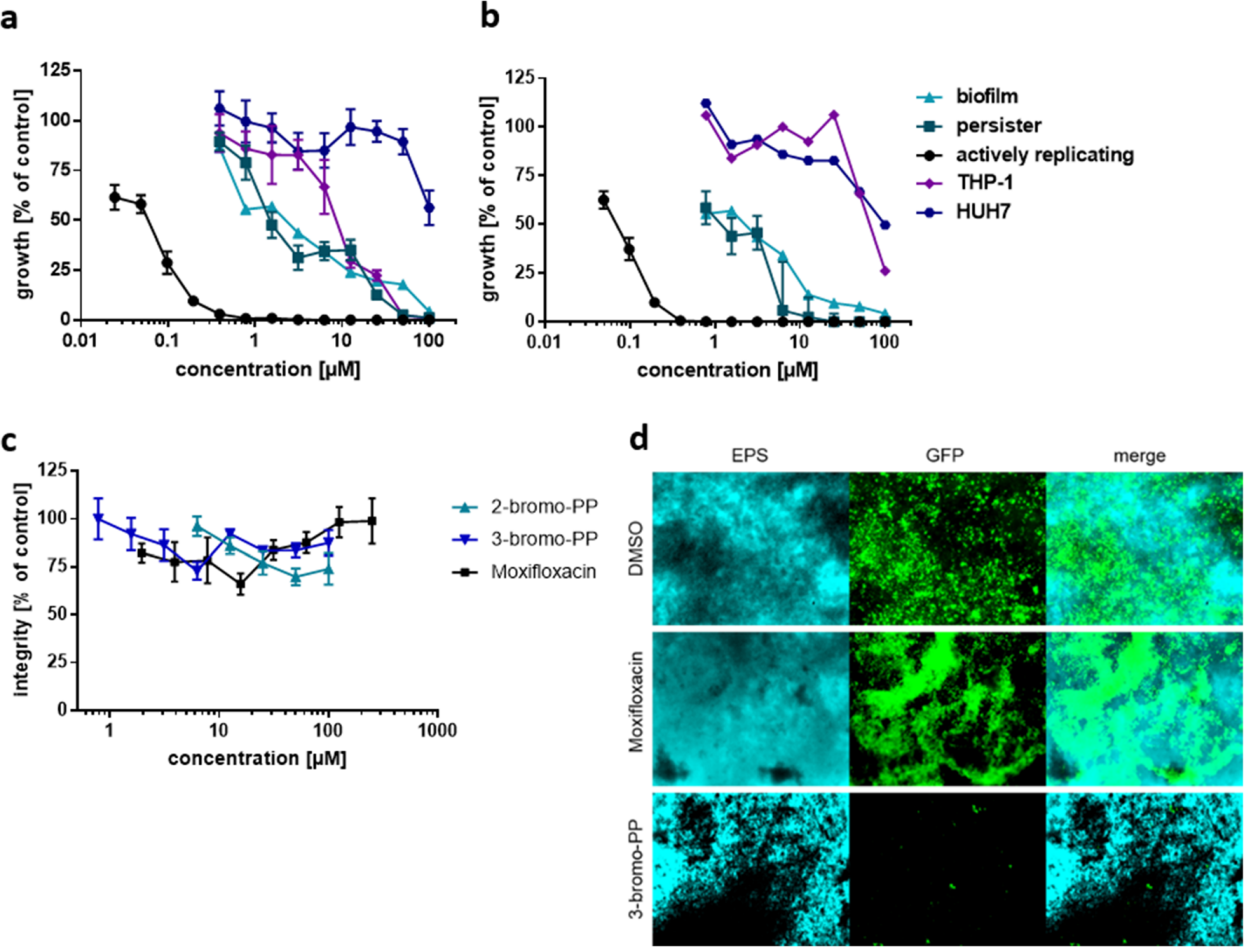
Antimicrobial activity of brominated phenoxyphenols. Minimal inhibitory concentrations and cytotoxicity data for **a** 2-bromo-PP and **b** 3-bromo-PP against actively replicating (black circle), dormant (turquoise square) and biofilm-incorporated (light blue triangle) MRSA cells. THP-1 monocytes (purple rhombus) and Huh7 hepatocytes (blue hexagon) were used to determine cytotoxicity. For persister, biofilm and eukaryotic viability assays, the Resazurin dye reduction assay was used. For actively replicating cells, the BacTiter Glo assay was used. To measure the biofilm integrity (**c**), crystal violet staining was used. Neither 2-bromo-PP (light blue triangle) nor 3-bromo-PP (blue inverted triangle) disperse biofilms. Moxifloxacin (black square) was used as control. Calculations were made with respect to the controls. Values are means of triplicates ± SEM. Fluorescence microscopy was used to visualise the impact of 3-bromo-PP on preformed biofilms (**d**). EPS was stained with Alcian blue; GFP was expressed by MRSA Mu50 GFP-reporter strain. DMSO and moxifloxacin were used as controls

In an initial screening with an in-house library of natural compounds, 2-bromo-PP and 3-bromo-PP have been found to inhibit growth of *S. aureus* and *P. aeruginosa*. Further experiments have been performed with both PPs in microbroth dilution assay for activity tests against different MRSA strains and various nosocomial pathogens, most representing the ESKAPE group (Table 1). In general, the activity of 3-bromo-PP was better against gram-positive (MIC_90_ ranging from 0.1 to 0.78 μM), whereas that of 2-bromo-PP was better against gram-negative bacteria (MIC_90_ ranging from 0.78 to 3.125 μM), including a multidrug-resistant clinical isolate of *E. cloacae* harbouring the metallo-ß-lactamase (MBL) bla_GIM-1_ as well as resistance genes against aminoglycosides, fluoroquinolones, thrimethoprin/sulfamethoxazole, tetracylines, tetracylins, fosmomycin and chloramphenicol (Wendel et al. 2013; Wendel et al. 2016). Activity of both compounds against gram-negative bacteria could be substantially increased when used in combination with a sublethal concentration of colistin (0.1 μM) to increase the permeability of the outer membrane (Table 1). Furthermore, PPs were tested against various human cell lines to assess their general cytotoxicity (Fig. 2a, b; Table S3). 2-Bromo-PP showed moderate cytotoxicity (IC_50_ = 6.25 μM) against THP-1 cells, but only minor cytotoxic effects against the other tested cell lines (IC_50_ = 12.5–> 100 μM). In contrast, virtually no cytotoxic effects were observed for 3-bromo-PP (IC_50_ = 25–> 100 μM) with the exception of HEK293 cells (IC_50_ = 3.125 μM). These data are in accordance with a previous study that reported on low cytotoxicity of both compounds for human PBMNCs (Mayer et al. 2019). Next, the activity of PPs was tested against persisters and preformed biofilms of MRSA Mu50 and of *P. aeruginosa* POA1. Both compounds showed activity against persisters with an MIC of 12.5 μM for 2-bromo-PP for both MRSA and *P. aeruginosa* as well as 6.25 μM and 50 μM for 3-bromo-PP against MRSA (Fig. 2a, b) and *P. aeruginosa* (Fig. S1a), respectively. For MRSA biofilms, 2-bromo-PP inhibited viability of cells in biofilms at 100 μM, while 3-bromo-PP was more potent and showed a maximal effect already at 12.5 μM as revealed by the resazurin reduction assay (Fig. 2a, b). For *P. aeruginosa* biofilms, 2-bromo-PP showed an increased effect compared with MRSA biofilms (complete inhibition at 25 μM), whereas 3-bromo-PP was less potent with complete inhibition of cell viability at 50 μM (Fig. S1b). Although both PPs showed a substantial effect on the viability of biofilm-incorporated cells, neither of the PPs influenced integrity of preformed biofilms as estimated by crystal violet staining (Fig. 2c) and fluorescence microscopy employing Alcian blue to stain the EPS (Fig. 2d).

**Table 1:** Activity of 2-bromo-PP and 3-bromo-PP against various nosocomial pathogens. The antimicrobial activity of PPs is notably good against multidrug-resistant bacteria. Asterisks indicate multidrug-resistant strains. The MIC of colistin against *A. baumannii* ATCC 747 and *E. coli* ATCC 25922 was 0.78 μM (0.90 μg/mL). The growth of gram-negative bacteria was not inhibited at a colistin concentration of 0.1 μM (0.12 μg/mL)

Organism	MIC_90_ μM (μg/mL)	
	2-Bromo-PP	3-Bromo-PP
*Staphylococcus aureus* strain ATCC 25923	0.39 (0.20)	0.78 (0.45)
*S. aureus* Mu50*	0.19 (0.10)	0.1 (0.06)
*S. aureus* COL*	0.78 (0.39)	0.78 (0.45)
*S. aureus* USA300*	0.78 (0.39)	0.39 (0.23)
*S. aureus* TCH1516*	0.78 (0.39)	0.78 (0.45)
*Enterococcus faecium* ATCC 35667	0.78 (0.39)	0.39 (0.23)
*E. faecium* ATCC 700221*	0.78 (0.39)	0.78 (0.45)
*E. faecalis* ATCC 29212	0.78 (0.39)	0.78 (0.45)
*E. faecalis* ATCC 51299*	0.78 (0.39)	0.78 (0.45)
*Bacillus subtilis* 168 trp C2	0.39 (0.20)	0.39 (0.23)
*Acinetobacter baumannii* ATCC 747	0.78 (0.39)	3.125 (1.81)
*A. baumannii* ATCC 1605*	0.78 (0.39)	1.56 (0.91)
*A. baumannii* ATCC 1605* + 0.1 μM colistin	0.39 (0.20)	0.78 (0.45)
*Pseudomonas aeruginosa* PAO1	0.78 (0.39)	1.56 (0.9)
*Escherichia coli* ATCC 25922	3.125 (1.57)	6.25 (3.63)
*Escherichia coli* ATCC 25922 + 0.1 μM colistin	0.04 (0.02)	0.04 (0.02)
*Klebsiella pneumoniae* ATCC 700603	1.56 (0.78)	3.125 (1.81)
*Enterobacter cloacae* isolate 3678*	12.5 (6.27)	12.5 (7.26)

### PPs have bactericidal activity against replicating, persistent and biofilm-incorporated MRSA cells

**Fig. 3:**
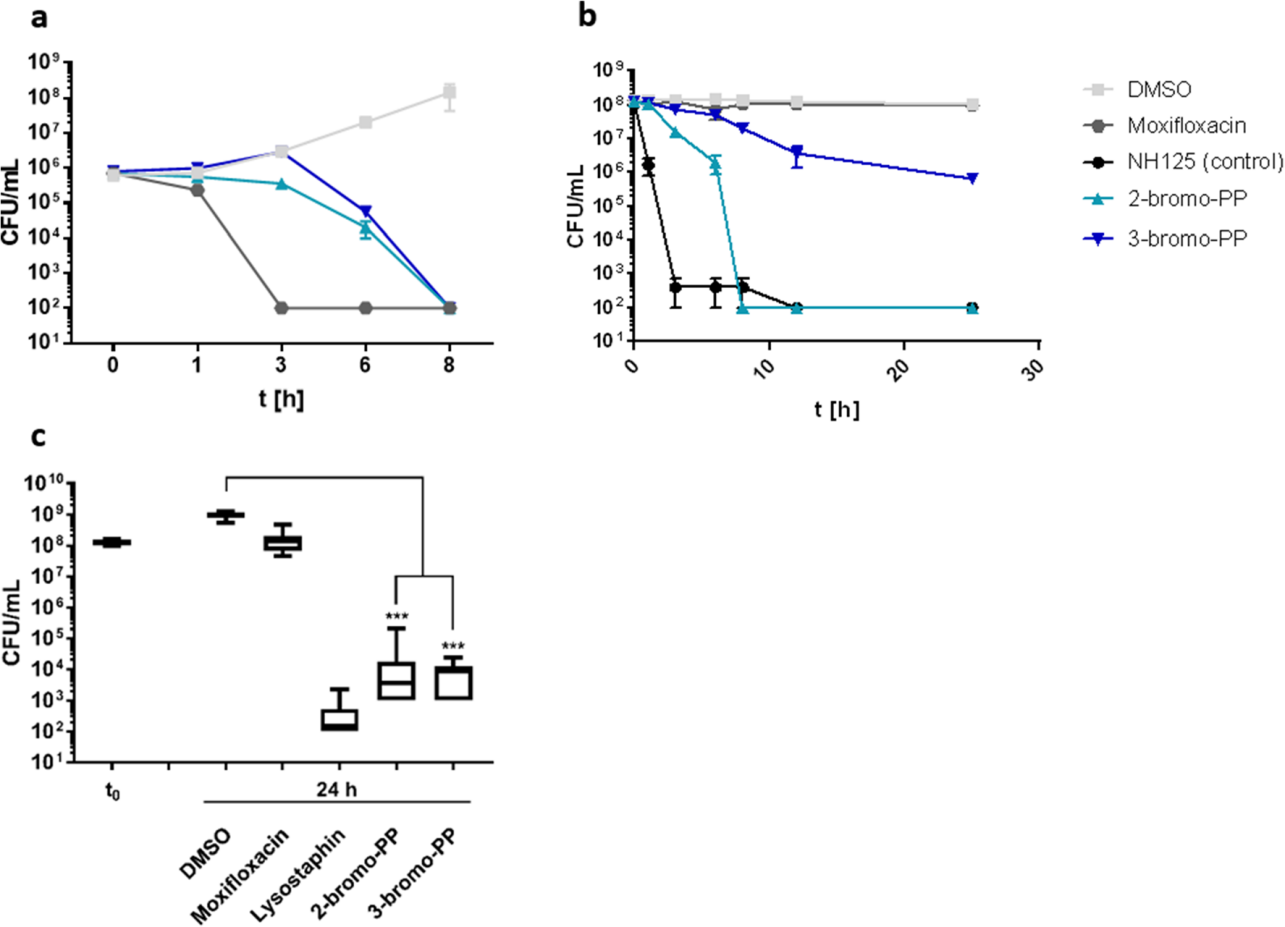
Bactericidal effects of 2-bromo-PP and 3-bromo-PP on cells of MRSA strain Mu50. Actively replicating (**a**) or stationary phase–induced persistent cells (**b**) were cultivated in MHB with the indicated compounds, and viability was monitored by CFU plating at various time points. Samples were inoculated at a starting cell density of 10^6^ CFU/mL (**a**) or 10^8^ CFU/mL (**b**), respectively. Limit of detection was 10^2^ CFU/mL. Values are means of triplicates ± SEM. Compounds have been added at the following concentrations: DMSO 50 μM (solvent control, light grey square); moxifloxacin 20 μM (positive control for actively replicating cells, grey rhombus); NH125 10 μM (positive control for persistent cells, black circle); 2-bromo-PP 0.76 μM (**a**) and 50 μM (**b**) (=4× MIC, light blue triangle); 3-bromo-PP 0.4 μM (**a**) and 25 μM (**b**) (=4× MIC, blue inverted triangle). **c** Antibiofilm activity of 2-bromo-PP and 3-bromo-PP against MRSA biofilms. Pre-formed MRSA biofilms were treated with 4× minimal biofilm inhibitory concentrations as calculated from the Resazurin reduction assay (see Fig. 2). After 24 h of treatment, biofilms were dispersed by ultrasonification and CFU plating was performed. Data represent means of *n* = 8 and were analysed using the Kolmogorov-Smirnov and Mann-Whitney *U* test. The limit of detection was 100 CFU/mL. Compounds have been added at the following concentrations: DMSO 50 μM (solvent control); moxifloxacin 20 μM (negative control); lysostaphin 25 μg/mL (positive control); 2-bromo-PP 50 μM; 3-bromo-PP 100 μM

For further characterization of PPs, killing kinetics were performed with cells of MRSA strain Mu50 cultivated in Mueller-Hinton broth (MHB) containing compounds at 4× MIC. Viability was monitored for 8 or 24 h for actively growing or stationary phase-induced persistent cells, respectively, by plating of serial dilutions and determinating colony-forming units (CFUs) (Fig. 3). For actively replicating MRSA, moxifloxacin as a positive control exhibited a strong and rapid bactericidal effect as expected with viability reaching the detection limit after 3 h of incubation. In contrast, 2-bromo-PP and 3-bromo-PP acted much slower with first antibacterial effects occuring after 3 and 6 h, respectively. Notwithstanding, both compounds also showed a slow but strong bactericidal effect, and viability eventually reached the detection limit after 8 h (Fig. 3a). Furthermore, a comparable bactericidal effect of both 2-bromo-PP and 3-bromo-PP was observable against replicating cells of multidrug-resistant *Acinetobacter baumannii* (Fig. S2).

In contrast to this potent bactericidal activity on actively growing MRSA cells, moxifloxacin did not show any effect against stationary phase-induced persistent cells even after 24 h of incubation. On the other hand, NH125, which has been reported to be active against persister cell populations (Kim et al. 2016), was used as a positive control in this assay and was able to reduce cell viability almost down to the detection limit after 3 h of incubation (Fig. 3b). Compared with NH125, 2-bromo-PP acted with a slight delay but eventually reduced viability dramatically down to the detection limit after 8 h of treatment. Thus, this compound is affecting both actively replicating and persister cells of MRSA, although substantially higher concentrations were required for achieving effects on persister cells. 3-Bromo-PP had a weaker effect on persistent cells and steadily decreased viability by 2 logs after 24 h (Fig. 3b). Additionally, 2-bromo-PP and 3-bromo-PP demonstrated strong bactericidal effects on MRSA biofilms in a CFU plating assay with reduction in viable cell counts by ca. 5 order of magnitudes after 24 h of treatment, whereas moxifloxacin again showed virtually no effect (Fig. 3c). However, although these data demonstrate that PPs provide activity both against planktonic cells as well as hard-to-treat subpopulations, we are aware that the antipersistence and antibiofilm activity of both compounds is much weaker compared with their potency against replicating cells. Thus, the therapeutic window for effective therapy against persisters and biofilm-incorporated cells is rather small.

### The bacterial phosphotransferase system is crucial for PP activity

**Fig. 4:**
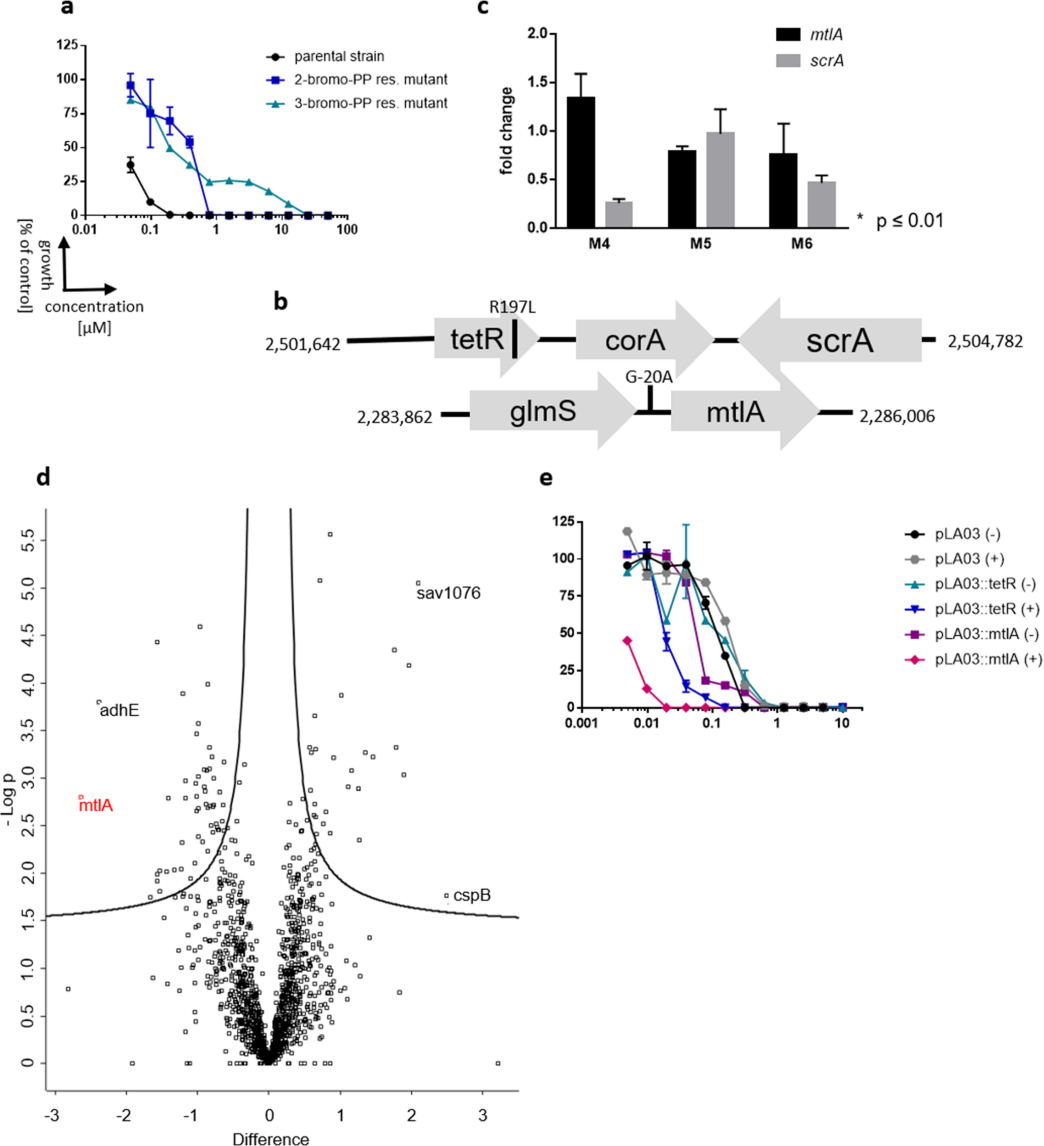
Analysis of spontaneously resistant *S. aureus* mutants. **a** Dose-response curve against 3-bromo-PP of the MRSA parental strain Mu50 (black circle), spontaneously resistant mutant M1 raised against 2-bromo-PP (blue square) and spontaneously resistant mutant M4 raised against 3-bromo-PP (turquoise triangle) showing a 4× and 32× MIC shift, respectively. Moreover, we observe cross-resistance between PPs. **b** The genomic regions of SRM containing mutations. SNPs are marked with a black line, and amino acid changes or nucleotide changes are annotated. **c** RT-qPCR of SRMs to check expression levels of *mtlA* (black) and *scrA* (grey). Expression was normalised to wild-type expression levels for each gene (set to 1). Statistical significance was determined using the Student’s *t* test. Error bars show standard error of mean; *n* = 3. **d** Proteome analysis of MRSA Mu50 cells treated with a sublethal concentration of 3-bromo-PP. Red marked protein MtlA is downregulated by approx. 2.5-fold. **e** Overexpression of *tetR* and *mtlA* render the cells more susceptible to 3-bromo-PP. Induced expression is indicated with a plus (grey hexagon, blue inverted triangle, magenta rhombus); non-induced expression (black circle, turquoise triangle, violet square) is depicted with a minus. The expression was induced using 1 μg/mL anhydrotetracycline

Spontaneous resistant mutants have been generated with 2-bromo-PP and 3-bromo-PP to further investigate the mechanism of antibacterial action and resistance. Since isolation of spontaneous resistant mutants in one step was not successful on solid media containing 2-bromo-PP or 3-bromo-PP (i.e. resistance frequency < 10^−7^ at 5-fold MIC), enrichment was done by passaging bacteria daily in liquid media containing increasing sublethal concentrations of PPs. After 15 passages, mutants were obtained that showed a 4- (2-bromo-PP) or 32-fold (3-bromo-PP) increased MIC, respectively (Table 2). Moreover, we observed cross-resistance between PPs since 2-bromo-PP-resistant mutants were also resistant against 3-bromo-PP and vice versa (Fig. 4a; Table 2).

**Table 2:** SNPs in spontaneously resistant mutants raised against 2- or 3-bromo-PP. *S. aureus* strain ATCC 25923 was used to generate resistant mutants against 2-bromo-PP, and *S. aureus* strain Mu50 was used to generate resistant mutants against 3-bromo-PP. A full list of identified SNPs can be found in Table S4

Strain	Parental strain	MIC_90_ 2-Bromo-PP μM (μg/mL)	MIC_90_ 3-Bromo-PP μM (μg/mL)	Relevant SNP
ATCC 25923	–	0.39 (0.20)	0.78 (0.45)	
Mu50	–	0.19 (0.10)	0.1 (0.06)	
M1	ATCC 25923	1.56 (0.78)	3.125 (1.81)	KQ76_12400: S155F (*tetR*)
M2	ATCC 25923	1.56 (0.78)	3.125 (1.81)	KQ76_12400: S155F (*tetR*)
M3	ATCC 25923	0.78 (0.39)	6.25 (3.63)	KQ76_12400: S155F (*tetR*)
M4	Mu50	1.56 (0.78)	3.125 (1.81)	SAV_RS12945: R197L (*tetR*)
M5	Mu50	1.56 (0.78)	3.125 (1.81)	C to G substitution between SAV_RS11755/11760 (5′ to *mtlA*)
M6	Mu50	0.78 (0.39)	3.125 (1.81)	SAV_RS12945: R197L (*tetR*)

Genomic DNA of six different resistant mutants raised against either 2-bromo-PP or 3-bromo-PP, respectively, was isolated, and genome sequencing was performed to analyse the mechanism of resistance. As a result of the serial sub-passaging at sublethal compound concentrations, the mutants had accumulated multiple genetic alterations as expected. However, most of these mutations occurred only once among the mutants, indicating that they were likely not relevant for resistance (Table S2). However, a SNP in the gene *tetR* coding for a transcription regulator was found in 5 of the 6 analysed mutants, strongly suggesting that this mutation is causally involved in the resistance phenotype (Table 2). In close chromosomal proximity to *tetR*, genes of the magnesium transporter *corA* and the phosphotransferase system (PTS) component *scrA* can be found (Fig. 4b). ScrA is predicted to be the EIIBC component of a sucrose-specific PTS (Deutscher et al. 2014; Wagner et al. 1993). Interestingly, one mutant (M5) harboured a SNP in the conceivable promoter region upstream of the *mtlA* gene as part of another PTS system (Fig. 4b). MtlA is an essential component of the mannitol-specific PTS (Reiche et al. 1988; UniProt 2019). These observations pointed toward a potential role of PTS systems in the resistance mechanisms.

In order to investigate how the mutations identified in the spontaneous resistant mutants affected expression levels of *scrA* and *mtlA*, qPCR analyses were performed. We found a ca. 0.75-fold decrease in the expression of *mtlA* in mutant M5 that carries a G to A substitution in the putative promotor region of *mtlA* (Fig. 4c). For mutant M4, which harbours a SNP in the *tetR* gene leading to an R197L amino acid exchange in the TetR protein (Table 2), we observed a strong and statistically significant downregulation of *scrA* expression (Fig. 4c). A similar trend was found for mutant M6, which carries an identical SNP, although differences in expression levels did not reach statistical significance (Fig. 4c).

For further investigation of the resistance mechanism against PPs, Mu50 cells were treated with a sublethal concentration of 3-bromo-PP that allowed ca. 50% residual growth compared with the untreated control. Subsequently, whole protein extracts were prepared to perform global proteome analysis of the elicited stress response. This revealed that MtlA was the most downregulated protein (approx. 2.5-fold) in sublethally 3-bromo-PP-stressed cells (Fig. 4d). In combination, these results suggest that reduced expression of EIIBC components of certain sugar-specific PTS such as MtlA or ScrA mediate resistance toward PPs.

To corroborate the role as potential determinants of resistance, *mtlA* and *tetR* were overexpressed under control of an anhydrotetracycline (Atc)-inducible promoter, and the effect on sensitivity toward 3-bromo-PP was analysed (Fig. 4e). The vector control strain harbouring the empty plasmid pLA03 showed an MIC of approx. 0.625 μM irrespective of the presence or absence of Atc. Non-induced strains containing the expression plasmids pLA03::*mtlA* and pLA03::*tetR* showed a marginal shift of the MIC to 0.3125 μM. However, induction of *mtlA* or *tetR* expression in the presence of Atc strongly rendered the cells more susceptible to 3-bromo-PP resulting in MICs of 0.019 μM for *mtlA* and 0.078 μM for *tetR* overexpression, respectively. This translates into a 33-fold and 8-fold increased sensitivity during *mtlA* or *tetR* overexpression, respectively, compared with the empty vector control (Fig. 4e). This characteristic differential susceptibility pattern in mutants and recombinant strains underexpressing or overexpressing *mtlA* or *tetR* strongly indicates that both genes are causally involved in the mechanism of resistance against PPs.

## Discussion

In this study, we demonstrated that both 2-bromo-PP and 3-bromo-PP are potent inhibitors of bacterial growth of various ESKAPE pathogens. Whereas 2-bromo-PP is more potent against gram-negative bacteria such as *P. aeruginosa* or *A. baumannii*, 3-bromo-PP is more active against gram-positive bacteria like MRSA. However, the most interesting feature of these compounds is their activity against persistent and biofilm-incorporated cells. The majority of antibiotics in clinical use show only good activity against actively replicating cells, while the available therapeutic options against persisters and especially biofilms are very limited (Hoiby et al. 2015). The effect on persistent bacteria is diminished because several antibiotics target processes that are downregulated during persistence or shut-off completely. Fluorchinolones such as moxifloxacin, for example, target the bacterial DNA-gyrase and have no activity against MRSA persisters at physiologic relevant concentrations (Drlica and Zhao 1997). This is also true for other entire families of antibiotics such as sulphonamides interfering indirectly with the nucleotide synthesis, and tetracyclines that are inhibiting protein biosynthesis (Chukwudi 2016; Henry 1943). Biofilm-incorporated cells, in particular, are even more tolerant to antibiotics although the biofilm is composed of persisters as well as actively replicating cells (Barraud et al. 2013). Thus, it is an important trait that PPs are not only killing gram-positive and gram-negative persisters, but also biofilm-incorporated cells in vitro.

The biofilm matrix is a strong physical barrier against large molecules like rifamycins and glycopeptides. This might be one reason for the high tolerance against these classes of antibiotics. Small molecules, such as PPs, are promising candidates to pass the complex biofilm matrix and to reach biological active concentrations inside the biofilm. In agreement with this, we observed a slow but constant reduction of viability so that eventually the majority of biofilm-incorporated bacteria were killed within 24 h by PPs. The biofilm matrix itself, however, was not affected in its integrity. In consequence, we could show that PPs are bactericidal against actively replicating MRSA Mu50 and *P. aeruginosa* cells as well as against their dormant counterparts and biofilm-incorporated cells.

During our studies, we were able to generate spontaneously resistant mutants performing liquid enrichment and genome sequencing to elucidate the mode of resistance. We found a consistent single nucleotide polymorphism (SNP) in several independent mutants in the transcription factor *tetR* resulting in an amino acid exchange (R197L). Interestingly, TetR appears to be a positive regulator of the neighbouring gene *scrA*, which is the EIIBC component of sucrose-specific PTS, since we found that the TetR^R197L^ mutation led to a lower expression of *scrA.* Furthermore, a G to A substitution in the putative promotor region of *mtlA* was found in one mutant that did not have the SNP in the *tetR* ORF. This promoter mutation led to a lower expression of *mtlA* as revealed by RT-qPCR analysis. MtlA is the EIIBC component of the mannitol-specific PTS. It catalyses the phosphorylation of incoming D-mannitol to D-mannitol-1-phosphate by translocating a phosphate group of an N-phospho-L-histidine residue to the sugar. D-mannitol-1-phosphate is then passed through the membrane. Likewise, ScrA is a sucrose-specific EIIBC PTS component that catalyses a similar reaction for sucrose. This strongly pointed toward a specific role of these EIIBC components and PTS in resistance to PPs. This hypothesis was further corroborated by the strong increase in sensitivity to 3-bromo-PP in recombinant MRSA strains overexpressing either *mtlA* or *tetR*, and by the strong downregulation of MtlA in cells treated with a sublethal concentration of 3-bromo-PP as revealed by proteome analysis. This differential susceptibility pattern (i.e. decreased sensitivity at reduced expression, increased sensitivity during overexpression) is in agreement with a potential role of the identified PTS for uptake of PPs. Based on the regular mechanism of EIIBC components in sugar uptake by PTS, it is conceivable that uptake of PPs is coupled to phosphorylation of the hydroxyl moiety in the left part of the molecule, potentially resulting in intracellular activation. Downregulation of MtlA in 3-bromo-PP treated cells might represent a specific stress response aiming at reducing the uptake of the antibacterial compound. Although we did not observe a similar stress response for ScrA, it is likely that both PTS have a redundant or additive role in uptake and/or activation of PPs. This might explain why we were unable to isolate single-step spontaneous PP-resistant mutants and obtained a low level of resistance only after serial subpassaging at escalating sublethal doses of the compounds. It is possible that further EIIBC PTS components might also be able to phosphorylate and subsequently take up PPs to some extent. Sofar, however, we have no evidence for the involvement of other PTS, e.g. the fructose PTS, in resistance to PPs.

With respect to the clinical potential of the studied PPs, a medicinal chemical optimisation regarding the mitigation of cytotoxic effects would be a major point besides the derivatization to increase the antibacterial potency. Most importantly, optimisation of the antibiofilm activity should be in the focus because the current therapeutical window is too small for proper clinical use by now. The synthesis of polybrominated diphenyl ethers (PBDEs), PP-like molecules, is well understood (Lin et al. 2014), which will greatly facilitate such medicinal chemistry efforts and will also support mode-of-action studies. For instance, synthesis of phosphorylated PPs could help to test the postulated requirement of phosphorylation by EIIBC components for activating PPs.

In summary, PPs are small bioactive molecules that are able to kill gram-positive and gram-negative bacteria. Most importantly, PPs kill not only planktonic bacterial cells but also their persistent counterparts, biofilm-incorporated and MDR enterobacteria cells as well. Some compounds are in development showing promising results in either preventing the attachment of bacteria to surfaces as the first step in biofilm formation (Elshaarawy et al. 2017) or the maturation of the biofilm via quorum quenching (Gopu et al. 2015), but these molecules are incapable to sterilise preformed biofilms. In this regard, PPs provide interesting new hit structures in the quest for new antibiofilm antibiotics. Advantages of PPs are their low potency to develop resistances (resistance rate < 10^−8^) as well as their broad spectrum activity against ESKAPE pathogens. To elucidate the mechanism of action, further experiments with derivatives of PPs are necessary in the future.

## Electronic supplementary material

ESM 1 (PDF 173 kb)

## References

[CR1] Allison KR, Brynildsen MP, Collins JJ (2011). Metabolite-enabled eradication of bacterial persisters by aminoglycosides. Nature.

[CR2] Balaban NQ (2011). Persistence: mechanisms for triggering and enhancing phenotypic variability. Curr Opin Genet Dev.

[CR3] Balaban NQ, Merrin J, Chait R, Kowalik L, Leibler S (2004). Bacterial persistence as a phenotypic switch. Science.

[CR4] Barraud N, Buson A, Jarolimek W, Rice SA (2013). Mannitol enhances antibiotic sensitivity of persister bacteria in *Pseudomonas aeruginosa* biofilms. PLoS One.

[CR5] Bigger JW (1944). Treatment of staphylococcal infections with penicillin by intermittent sterilisation. Lancet.

[CR6] Boswihi SS, Udo EE (2018). Methicillin-resistant *Staphylococcus aureus* : an update on the epidemiology, treatment options and infection control. Current Medicine Research and Practice.

[CR7] Chambers HF, Deleo FR (2009). Waves of resistance: *Staphylococcus aureus* in the antibiotic era. Nat Rev Microbiol.

[CR8] Christensen GD, Simpson WA, Younger JJ, Baddour LM, Barrett FF, Melton DM, Beachey EH (1985). Adherence of coagulase-negative staphylococci to plastic tissue culture plates: a quantitative model for the adherence of staphylococci to medical devices. J Clin Microbiol.

[CR9] Chukwudi CU (2016). rRNA binding sites and the molecular mechanism of action of the tetracyclines. Antimicrob Agents Chemother.

[CR10] CLSI (2012) Methods for dilution antimicrobial susceptibility tests for bacteria that grow aerobically: approved standard Clinical and Laboratory Standarts Institute 9

[CR11] Conlon BP, Rowe SE, Gandt AB, Nuxoll AS, Donegan NP, Zalis EA, Clair G, Adkins JN, Cheung AL, Lewis K (2016) Persister formation in *Staphylococcus aureus* is associated with ATP depletion. Nat Microbiol 1(5). 10.1038/nmicrobiol.2016.51 10.1038/nmicrobiol.2016.51 PMC4932909 27572649

[CR12] Cox J, Hein MY, Luber CA, Paron I, Nagaraj N, Mann M (2014). Accurate proteome-wide label-free quantification by delayed normalization and maximal peptide ratio extraction, termed MaxLFQ. Mol Cell Proteomics.

[CR13] Cox J, Mann M (2008). MaxQuant enables high peptide identification rates, individualized p.p.b.-range mass accuracies and proteome-wide protein quantification. Nat Biotechnol.

[CR14] Cox J, Neuhauser N, Michalski A, Scheltema RA, Olsen JV, Mann M (2011). Andromeda: a peptide search engine integrated into the MaxQuant environment. J Proteome Res.

[CR15] Deutscher J, Ake FM, Derkaoui M, Zebre AC, Cao TN, Bouraoui H, Kentache T, Mokhtari A, Milohanic E, Joyet P (2014). The bacterial phosphoenolpyruvate:carbohydrate phosphotransferase system: regulation by protein phosphorylation and phosphorylation-dependent protein-protein interactions. Microbiol Mol Biol Rev.

[CR16] Drlica K, Zhao X (1997). DNA gyrase, topoisomerase IV, and the 4-quinolones. Microbiol Mol Biol Rev.

[CR17] Elshaarawy RFM, Mustafa FHA, van Geelen L, Abou-Taleb AEA, Tadros HRZ, Kalscheuer R, Janiak C (2017). Mining marine shell wastes for polyelectrolyte chitosan anti-biofoulants: fabrication of high-performance economic and ecofriendly anti-biofouling coatings. Carbohydr Polym.

[CR18] Flemming HC (2016) EPS—then and now. Microorganisms 4(4). 10.3390/microorganisms4040041 10.3390/microorganisms4040041 PMC5192524 27869702

[CR19] Foster TJ (2017). Antibiotic resistance in *Staphylococcus aureus*. Current status and future prospects. FEMS Microbiol Rev.

[CR20] Fu X, Schmitz FJ (1996). New brominated diphenyl ether from an unidentified species of *Dysidea* sponge. 13C NMR data for some brominated diphenyl ethers. J Nat Prod.

[CR21] Gopu V, Meena CK, Shetty PH (2015). Quercetin influences quorum sensing in food borne bacteria: in-vitro and in-silico evidence. PLoS One.

[CR22] Harkins CP, Pichon B, Doumith M, Parkhill J, Westh H, Tomasz A, de Lencastre H, Bentley SD, Kearns AM, Holden MTG (2017). Methicillin-resistant *Staphylococcus aureus* emerged long before the introduction of methicillin into clinical practice. Genome Biol.

[CR23] Hartman BJ, Tomasz A (1984). Low-affinity penicillin-binding protein associated with beta-lactam resistance in *Staphylococcus aureus*. J Bacteriol.

[CR24] Haysom L, Cross M, Anastasas R, Moore E, Hampton S (2018). Prevalence and risk factors for methicillin-resistant *Staphylococcus aureus* (MRSA) infections in custodial populations: a systematic review. J Correct Health Care.

[CR25] Henry RJ (1943). The mode of action of sulfonamides. Bacteriol Rev.

[CR26] Hoiby N, Bjarnsholt T, Givskov M, Molin S, Ciofu O (2010). Antibiotic resistance of bacterial biofilms. Int J Antimicrob Agents.

[CR27] Hoiby N, Bjarnsholt T, Moser C, Bassi GL, Coenye T, Donelli G, Hall-Stoodley L, Hola V, Imbert C, Kirketerp-Moller K, Lebeaux D, Oliver A, Ullmann AJ, Williams C, Biofilms ESGf, Consulting External Expert Werner Z (2015). ESCMID guideline for the diagnosis and treatment of biofilm infections 2014. Clin Microbiol Infect.

[CR28] Ioerger TR, Feng Y, Ganesula K, Chen X, Dobos KM, Fortune S, Jacobs WR, Mizrahi V, Parish T, Rubin E, Sassetti C, Sacchettini JC (2010). Variation among genome sequences of H37Rv strains of *Mycobacterium tuberculosis* from multiple laboratories. J Bacteriol.

[CR29] Jayaweera J, Kumbukgolla WW (2017). Antibiotic resistance patterns of methicillin-resistant *Staphylococcus aureus* (MRSA) isolated from livestock and associated farmers in Anuradhapura, Sri Lanka. Germs.

[CR30] Jevons MP (1961). “Celbenin”-resistant staphylococci. Bmj.

[CR31] Kaur DC, Chate SS (2015). Study of antibiotic resistance pattern in methicillin resistant *Staphylococcus aureus* with special reference to newer antibiotic. J Glob Infect Dis.

[CR32] Kim W, Fricke N, Conery AL, Fuchs BB, Rajamuthiah R, Jayamani E, Vlahovska PM, Ausubel FM, Mylonakis E (2016). NH125 kills methicillin-resistant *Staphylococcus aureus* persisters by lipid bilayer disruption. Future Med Chem.

[CR33] Kirby WM (1944). Extraction of a highly potent penicillin inactivator from penicillin resistant staphylococci. Science.

[CR34] Klein EY, Van Boeckel TP, Martinez EM, Pant S, Gandra S, Levin SA, Goossens H, Laxminarayan R (2018). Global increase and geographic convergence in antibiotic consumption between 2000 and 2015. Proc Natl Acad Sci U S A.

[CR35] Krausz KL, Bose JL (2016). Rapid isolation of DNA from *Staphylococcus*. Methods Mol Biol.

[CR36] Kurtz S, Phillippy A, Delcher AL, Smoot M, Shumway M, Antonescu C, Salzberg SL (2004). Versatile and open software for comparing large genomes. Genome Biol.

[CR37] Lakhundi S, Zhang K (2018) Methicillin-resistant *Staphylococcus aureus*: molecular characterization, evolution, and epidemiology. Clin Microbiol Rev 31(4). 10.1128/CMR.00020-18 10.1128/CMR.00020-18 PMC6148192 30209034

[CR38] Lewis AM, Rice KC (2016). Quantitative real-time PCR (qPCR) workflow for analyzing *Staphylococcus aureus* gene expression. Methods Mol Biol.

[CR39] Li H, Durbin R (2009). Fast and accurate short read alignment with Burrows-Wheeler transform. Bioinformatics.

[CR40] Lin K, Gan J, Liu W (2014). Production of hydroxylated polybrominated diphenyl ethers from bromophenols by bromoperoxidase-catalyzed dimerization. Environ Sci Technol.

[CR41] Lowy FD (1998). *Staphylococcus aureus* infections. N Engl J Med.

[CR42] Matsuhashi M, Song MD, Ishino F, Wachi M, Doi M, Inoue M, Ubukata K, Yamashita N, Konno M (1986). Molecular cloning of the gene of a penicillin-binding protein supposed to cause high resistance to beta-lactam antibiotics in *Staphylococcus aureus*. J Bacteriol.

[CR43] Mayer S, Prechtl M, Liebfried P, Cadeddu RP, Stuhldreier F, Kohl M, Wenzel F, Stork B, Wesselborg S, Proksch P, Germing U, Haas R, Jager P (2019) First results from a screening of 300 naturally occurring compounds: 4,6-dibromo-2-(2′,4′-dibromophenoxy)phenol, 4,5,6-tribromo-2-(2′,4′-dibromophenoxy)phenol, and 5-epi-nakijinone Q as substances with the potential for anticancer therapy. Mar Drugs 17(9). 10.3390/md17090521 10.3390/md17090521 PMC6780284 31491907

[CR44] Mermel LA, Allon M, Bouza E, Craven DE, Flynn P, O’Grady NP, Raad II, Rijnders BJ, Sherertz RJ, Warren DK (2009). Clinical practice guidelines for the diagnosis and management of intravascular catheter-related infection: 2009 update by the Infectious Diseases Society of America. Clin Infect Dis.

[CR45] Michalski A, Damoc E, Lange O, Denisov E, Nolting D, Muller M, Viner R, Schwartz J, Remes P, Belford M, Dunyach JJ, Cox J, Horning S, Mann M, Makarov A (2012) Ultra high resolution linear ion trap Orbitrap mass spectrometer (Orbitrap Elite) facilitates top down LC MS/MS and versatile peptide fragmentation modes. Mol cell proteomics 11(3):O111 013698 doi:10.1074/mcp.O111.013698 10.1074/mcp.O111.013698 PMC3316736 22159718

[CR46] Olsen JV, de Godoy LMF, Li GQ, Macek B, Mortensen P, Pesch R, Makarov A, Lange O, Horning S, Mann M (2005). Parts per million mass accuracy on an orbitrap mass spectrometer via lock mass injection into a C-trap. Mol Cell Proteomics.

[CR47] Prax M, Mechler L, Weidenmaier C, Bertram R (2016). Glucose augments killing efficiency of daptomycin challenged *Staphylococcus aureus* persisters. PLoS One.

[CR48] Rammelkamp CH, Maxon T (1942). Resistance of *Staphylococcus aureus* to the action of penicillin. Exp Biol Med.

[CR49] Rappsilber J, Mann M, Ishihama Y (2007). Protocol for micro-purification, enrichment, pre-fractionation and storage of peptides for proteomics using StageTips. Nat Protoc.

[CR50] Reiche B, Frank R, Deutscher J, Meyer N, Hengstenberg W (1988). Staphylococcal phosphoenolpyruvate-dependent phosphotransferase system: purification and characterization of the mannitol-specific enzyme IIImtl of *Staphylococcus aureus* and *Staphylococcus carnosus* and homology with the enzyme IImtl of *Escherichia coli*. Biochemistry.

[CR51] Stepanovic S, Vukovic D, Hola V, Di Bonaventura G, Djukic S, Cirkovic I, Ruzicka F (2007). Quantification of biofilm in microtiter plates: overview of testing conditions and practical recommendations for assessment of biofilm production by staphylococci. APMIS.

[CR52] Stewart B, Rozen DE (2012). Genetic variation for antibiotic persistence in *Escherichia coli*. Evolution.

[CR53] Tyanova S, Temu T, Sinitcyn P, Carlson A, Hein MY, Geiger T, Mann M, Cox J (2016). The Perseus computational platform for comprehensive analysis of (prote)omics data. Nat Methods.

[CR54] UniProt C (2019). UniProt: a worldwide hub of protein knowledge. Nucleic Acids Res.

[CR55] van Geelen L, Meier D, Rehberg N, Kalscheuer R (2018). (Some) current concepts in antibacterial drug discovery. Appl Microbiol Biotechnol.

[CR56] Vizcaino JA, Csordas A, del Toro N, Dianes JA, Griss J, Lavidas I, Mayer G, Perez-Riverol Y, Reisinger F, Ternent T, Xu QW, Wang R, Hermjakob H (2016). 2016 update of the PRIDE database and its related tools. Nucleic Acids Res.

[CR57] Wagner E, Gotz F, Bruckner R (1993). Cloning and characterization of the scrA gene encoding the sucrose-specific enzyme II of the phosphotransferase system from *Staphylococcus xylosus*. Mol Gen Genet.

[CR58] Wendel AF, Brodner AH, Wydra S, Ressina S, Henrich B, Pfeffer K, Toleman MA, Mackenzie CR (2013). Genetic characterization and emergence of the metallo-beta-lactamase GIM-1 in *Pseudomonas* spp. and *Enterobacteriaceae* during a long-term outbreak. Antimicrob Agents Chemother.

[CR59] Wendel AF, Ressina S, Kolbe-Busch S, Pfeffer K, MacKenzie CR (2016). Species diversity of environmental GIM-1-producing bacteria collected during a long-term outbreak. Appl Environ Microbiol.

[CR60] Wolk DM, Struelens MJ, Pancholi P, Davis T, Della-Latta P, Fuller D, Picton E, Dickenson R, Denis O, Johnson D, Chapin K (2009). Rapid detection of *Staphylococcus aureus* and methicillin-resistant *S. aureus* (MRSA) in wound specimens and blood cultures: multicenter preclinical evaluation of the Cepheid Xpert MRSA/SA skin and soft tissue and blood culture assays. J Clin Microbiol.

[CR61] Zipperer A, Konnerth MC, Laux C, Berscheid A, Janek D, Weidenmaier C, Burian M, Schilling NA, Slavetinsky C, Marschal M, Willmann M, Kalbacher H, Schittek B, Brotz-Oesterhelt H, Grond S, Peschel A, Krismer B (2016). Human commensals producing a novel antibiotic impair pathogen colonization. Nature.

