# Correction to: Natural brominated phenoxyphenols kill persistent and biofilm-incorporated cells of MRSA and other pathogenic bacteria

**DOI:** 10.1007/s00253-021-11409-5

**Published:** 2021-07-19

**Authors:** Lasse van Geelen, Farnusch Kaschani, Shabnam S. Sazzadeh, Emmanuel T. Adeniyi, Dieter Meier, Peter Proksch, Klaus Pfeffer, Markus Kaiser, Thomas R. Ioerger, Rainer Kalscheuer

**Affiliations:** 1 Institute of Pharmaceutical Biology and Biotechnology, Heinrich Heine University Düsseldorf, Dusseldorf, Germany; 2 Center of Medical Biotechnology, Chemical Biology, University Duisburg-Essen, Duisburg, Germany; 3 Institute of Medical Microbiology and Hospital Hygiene, Heinrich Heine University Düsseldorf, Dusseldorf, Germany; 4 Department of Computer Science, Texas A&M University, College Station, TX USA

**Correction to: Applied Microbiology and Biotechnology (2020) 104:5985–5998**

10.1007/s00253-020-10654-4

The article “Natural brominated phenoxyphenols kill persistent and biofilm-incorporated cells of MRSA and other pathogenic bacteri”, written by Lasse van Geelen, Farnusch Kaschani, Shabnam S. Sazzadeh, Emmanuel T. Adeniyi, Dieter Meier, Peter Proksch, Klaus Pfeffer, Markus Kaiser, Thomas R. Ioerger and Rainer Kalscheuer, was originally published Online First without Open Access. After publication in volume 104, issue 13, page 5985–5998 the author decided to opt for Open Choice and to make the article an Open Access publication. Therefore, the copyright of the article has been changed to © The Author(s) 2020 and the article is forthwith distributed under the terms of the Creative Commons Attribution 4.0 International License, which permits use, sharing, adaptation, distribution and reproduction in any medium or format, as long as you give appropriate credit to the original author(s) and the source, provide a link to the Creative Commons license, and indicate if changes were made. The images or other third party material in this article are included in the article’s Creative Commons license, unless indicated otherwise in a credit line to the material. If material is not included in the article’s Creative Commons license and your intended use is not permitted by statutory regulation or exceeds the permitted use, you will need to obtain permission directly from the copyright holder. To view a copy of this license, visit http://creativecommons.org/licenses/by/4.0. Open access funding enabled and organized by Projekt DEAL.

The original article was corrected.

