# Supplementary material for: Natural brominated phenoxyphenols kill persistent and biofilm-incorporated cells of MRSA and other pathogenic bacteria
Source: Appl Microbiol Biotechnol. 2020 May 16;104(13):5985–98. doi: 10.1007/s00253-020-10654-4 (PMC8217011; doi:10.1007/s00253-020-10654-4)
Supplement: Supplementary file 1 — (PDF 173 kb) [file 253_2020_10654_MOESM1_ESM.pdf]

Applied Microbiology and Biotechnology

Supplementary Data

Natural brominated phenoxyphenols kill persistent and biofilm-incorporated cells of pathogenic bacteria

Lasse van Geelen[1], Farnusch Kaschani[2], Shabnam S. Sazzadeh[3], Emmanuel T. Adeniyi[1], Dieter Meier[1], Peter Proksch[1], Klaus Pfeffer[3], Markus Kaiser[2], Thomas R. Ioerger[4], and Rainer Kalscheuer[1,*]

[1] Institute of Pharmaceutical Biology and Biotechnology, Heinrich Heine University Düsseldorf, Germany

[2] Center of Medical Biotechnology, Chemical Biology, University Duisburg-Essen, Germany

[3] Institute of Medical Microbiology and Hospital Hygiene, Heinrich Heine University Düsseldorf, Germany

[4] Department of Computer Science, Texas A&M University, College Station, Texas, USA

* Correspondence: Rainer Kalscheuer (rainer.kalscheuer@hhu.de; +49 211 81 14180)

Table S1 – Strains used in this study

Strain	Relevant properties	Origin
Staphylococcus aureus Mu50 (ATCC 700699)	Multidrug-resistant screening strain, parental strain for spontaneously resistant mutants M4 - M6	American Type Culture Collection (ATCC)
S. aureus Mu50 + pLA02::GFP	Constitutively expressing GFP-reporter strain	This study
S. aureus USA300	Multidrug-resistant screening strain	Biodefense and Emerging Infections Research Resources Repository (BEI Resources)
S. aureus TCH1516	Multidrug-resistant screening strain	BEI Resources
S. aureus COL	Multidrug-resistant screening strain	BEI Resources
S. aureus ATCC 25923	Screening strain, parental strain for spontaneously resistant mutants M1 - M3	ATCC
S. aureus RN4220	Cloning intermediate for *S. aureus* plasmids	German Collection of Microorganisms and Cell Cultures GmbH (DSMZ)
Enterococcus faecium ATCC 35667	Screening strain	ATCC
E. faecium ATCC 700221	Multidrug-resistant screening strain	ATCC
E. faecalis ATCC 29212	Screening strain	ATCC
E. faecalis ATCC 51299	Multidrug-resistant screening strain	ATCC
Bacillus subtilis 168 trp C2	Screening strain	ATCC
Acinetobacter baumannii ATCC 747	Screening strain	ATCC
A. baumannii ATCC 1605	Multidrug-resistant screening strain	ATCC
Enterobacter cloacae isolate 3678	Multidrug-resistant screening strain	Clinical isolate [16,17]
Klebsiella pneumonia ATCC 700603	Screening strain	ATCC
P. aeruginosa PAO 1	Screening strain, does not produce alginate	DSMZ
Escherichia coli ATCC 25922	Multidrug-resistant screening strain	ATCC
E. coli NEB 5-alpha	Cloning strain for plasmids	New England Biolabs (Cat.-No. C2987I)

Table S2 – Plasmids used in this study

Plasmid	Properties
pLA03::-	Chloramphenicol resistance, Atc-inducible expression vector for *S. aureus*
pLA03::tetR	Overexpression plasmid for *tetR*
pLA03::mtlA	Overexpression plasmid for *mtlA*
pLA02::GFP	Chloramphenicol resistance (cmR), GFP-reporter plasmid, generated from pCN57 [61] by ligation of cmR into *Apa*I- and *Xho*I-cut plasmid

Table S3 - Cytotoxicity data and cell lines

Cell line	IC_{50} [µM]	
	2- bromo-PP	3- bromo-PP
THP-1 (ATCC TIB-202)	6.25	50
MRC-5 (ATCC CCL-171)	>100	100
HEK293 (CLS 300192)	12.5	3.125
HepG2 (CLS 300198)	50	25
CLS-54 (CLS 300227)	>100	>100
HuH7 (CLS 300156)	>100	>100

Table S4 – Single nucleotide polymorphisms (SNPs) in spontaneously resistant mutants

Strain	SNPs
M1	KQ76_12400:S155F
M2	KQ76_12400:S155F
M3	KQ76_12400:S155F
M4	SAV_RS00960:G304W, SAV_RS05415:c-47a, SAV_RS05435:L182H, SAV_RS07330:C204*, SAV_RS07940:L96L, SAV_RS12945:R197L, SAV_RS14075:D296E, SAV_RS14630/hisZ:a-80t
M5	SAV_RS00525:S400F, SAV_RS01095:V461L, SAV_RS03035:G44V, SAV_RS07965:D209N, SAV_RS07970:S265F, SAV_RS13840:V23L, c>g between SAV_RS11755/11760
M6	SAV_RS00960:G304W, SAV_RS05415:c-47a, SAV_RS05435:L182H, SAV_RS07330:C204*, SAV_RS07940:L96L, SAV_RS12945:R197L, SAV_RS14075:D296E, SAV_RS14630/hisZ:a-80t

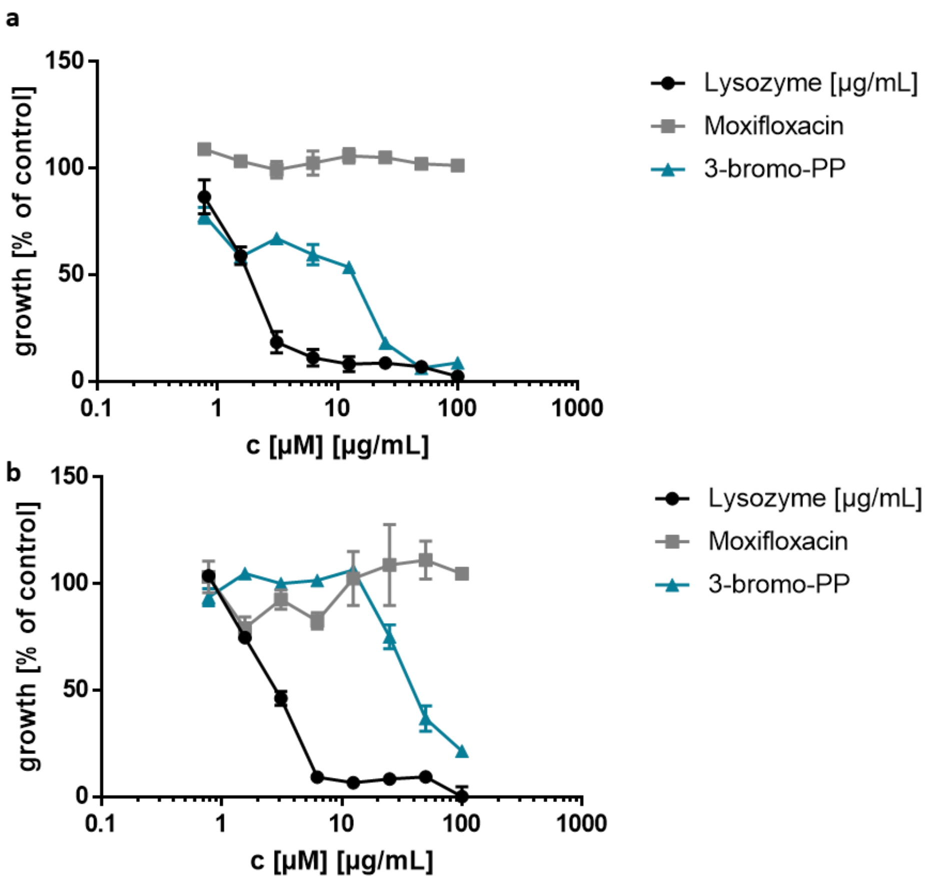

Figure S1 - 3-bromo-PP activity against *P. aeruginosa* PAO1 (a) persister and (b) biofilm. Lysozyme has been used as a positive and moxifloxacin as a negative control, respectively. 3-bromo-PP shows activity against *P. aeruginosa* PAO1 persisters and biofilm, with slightly weaker effects compared to MRSA Mu50 persisters and biofilm (see Figure 2).

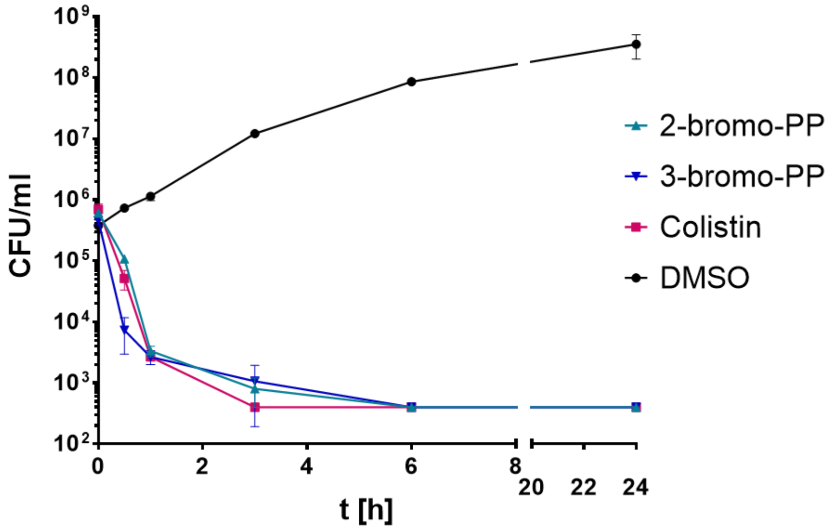

Figure S2 – Bactericidal effects of 2-bromo-PP and 3-bromo-PP against replicating cells of *A. baumannii* ATCC 747. Colistin was used as positive control (3.125 µM). 2-bromo-PP and 3-bromo-PP were used at concentrations of 3.125 µM and 6.25 µM, respectively. The limit of detection was 500 CFU/mL.
